# Supplementary figures and images for: Arthropod community composition in urban landscapes is shaped by both environmental filtering and dispersal limitation
Source: PLoS One. 2024 Aug 7;19(8):e0297507. doi: 10.1371/journal.pone.0297507 (PMC11305576; doi:10.1371/journal.pone.0297507)

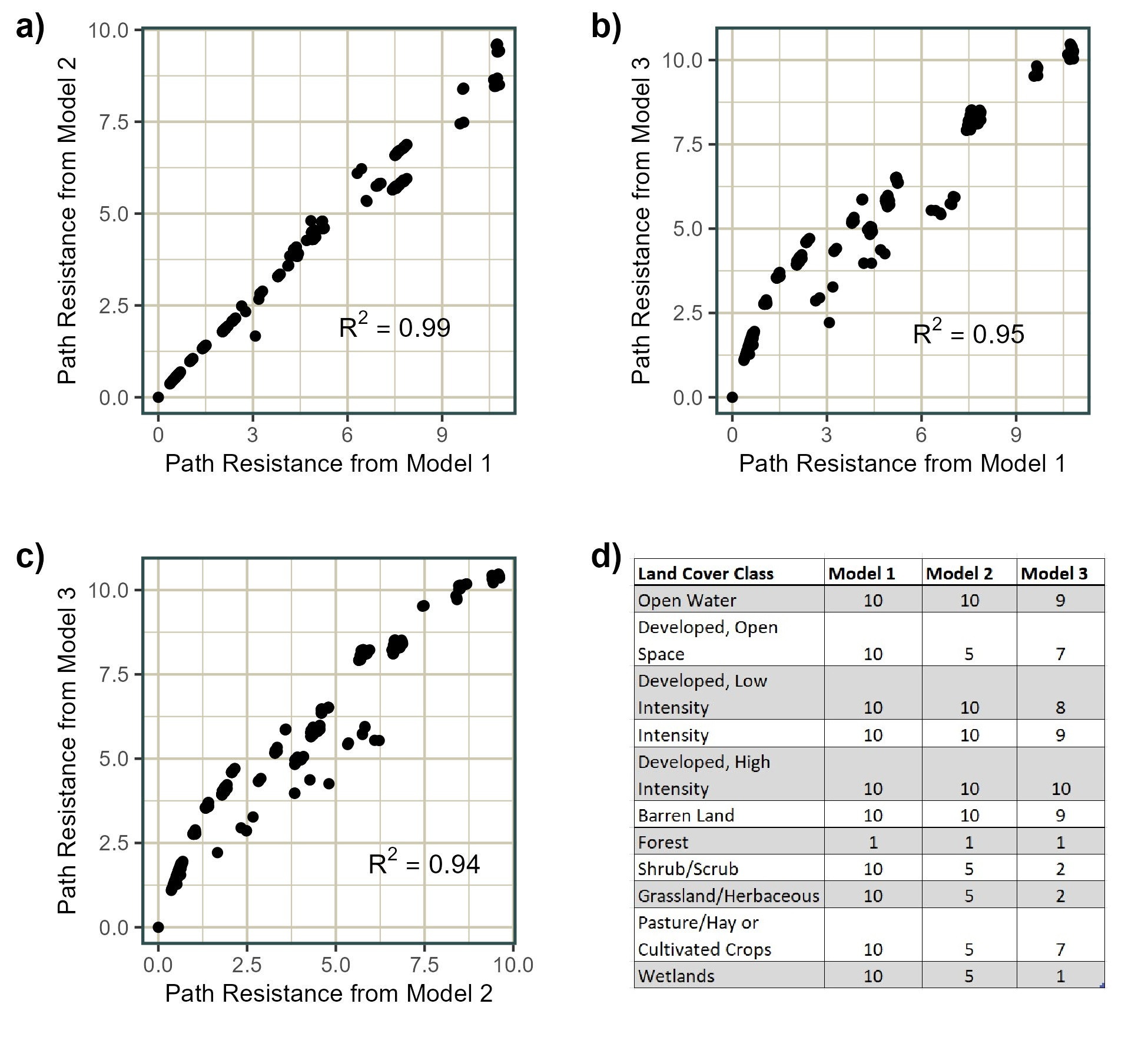

Supplement: S1 Fig — Scatterplots (a-c) of estimates of resistance of the shortest path between sampling plots using three different models of (d) how resistance values vary with land cover classes from the National Land Cover Database. Low resistance implies a species can disperse easily across that land cover type while high values imply the land cover type is a barrier to dispersal. (TIF) [file pone.0297507.s001.tif]

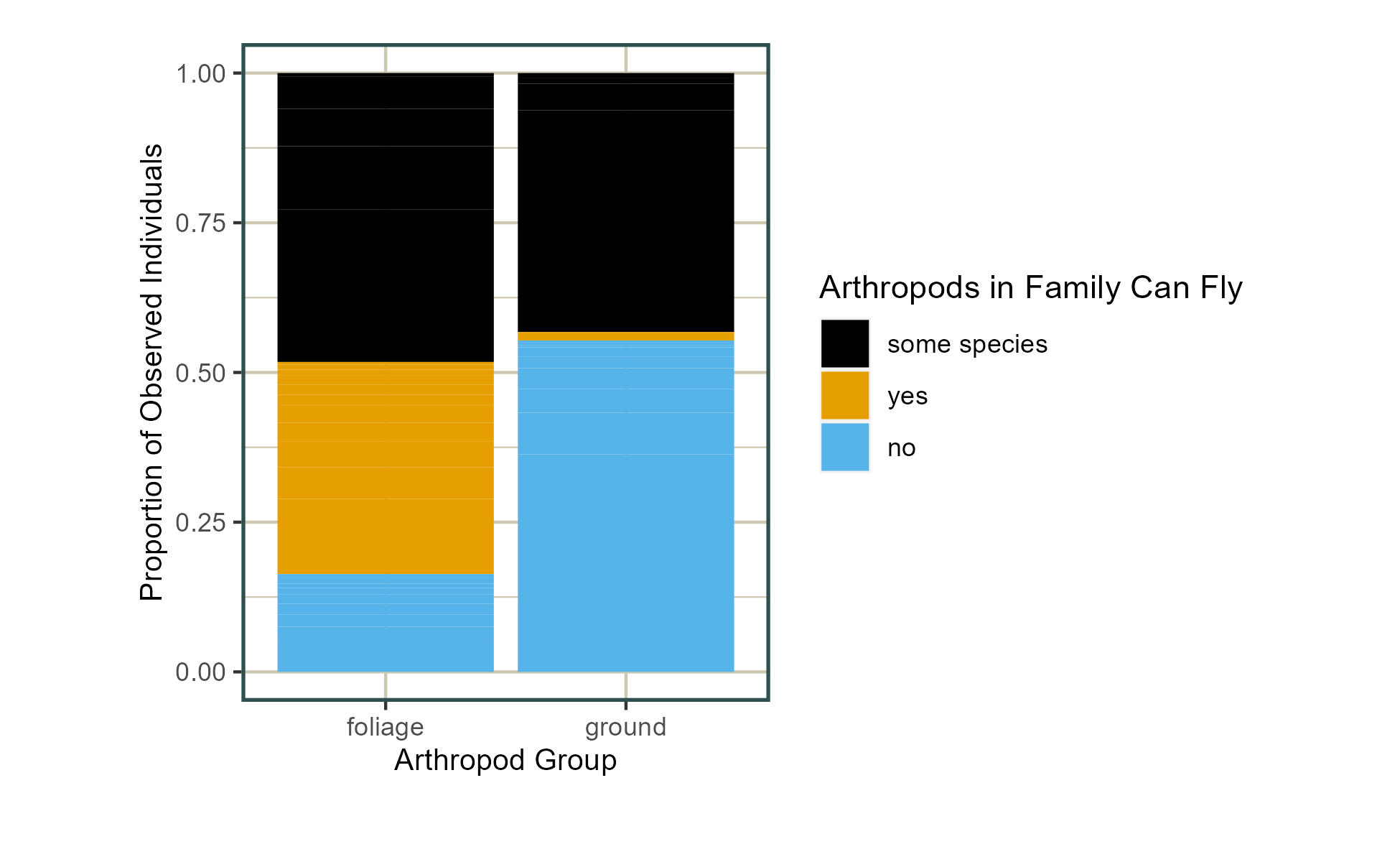

Supplement: S2 Fig — (TIF) [file pone.0297507.s002.tif]

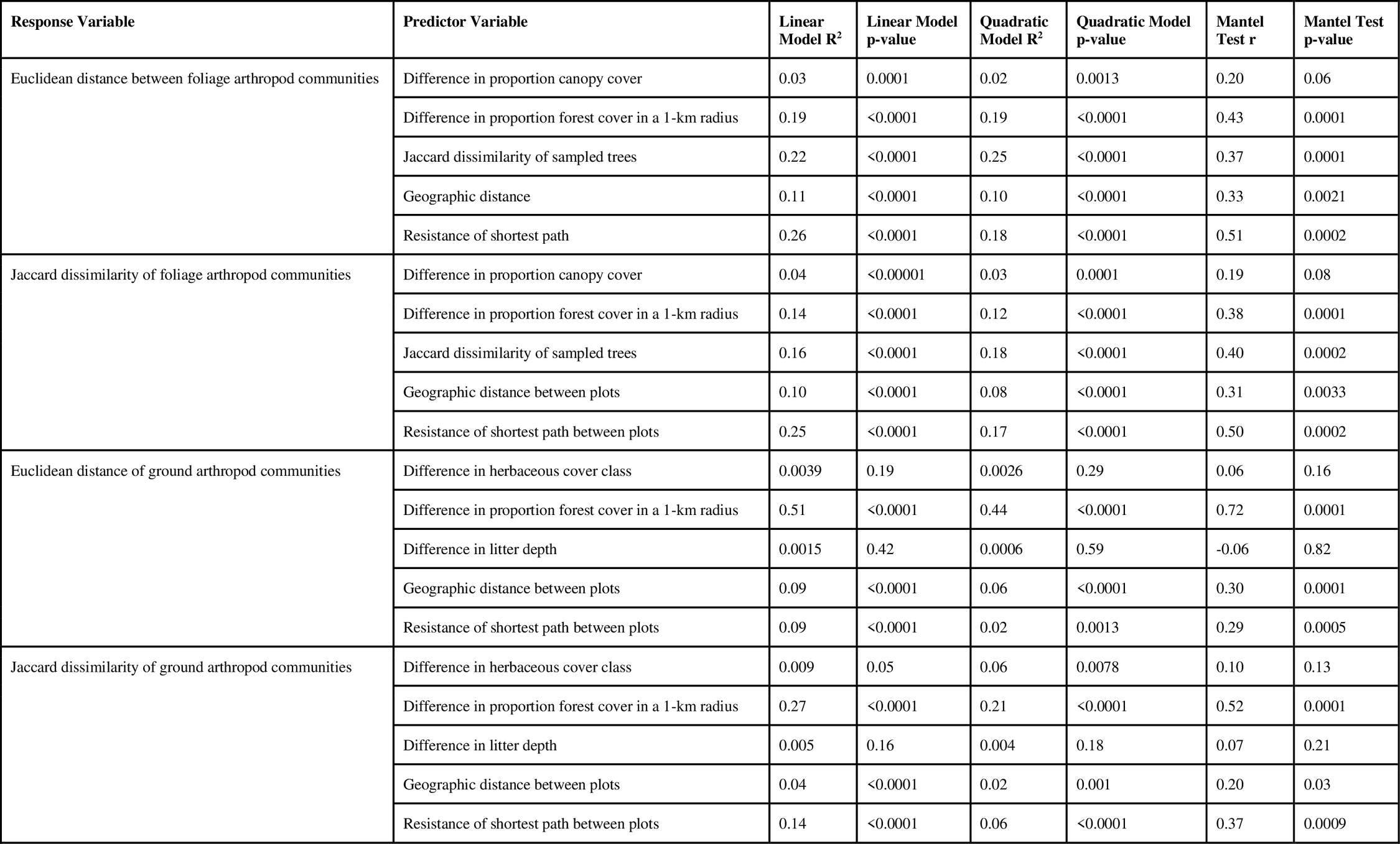

Supplement: S1 Table — (TIF) [file pone.0297507.s003.tif]
